# Social Determinants Predict Outcomes in Data From a Multi-Ethnic Cohort of 20,899 Patients Investigated for COVID-19

**DOI:** 10.3389/fpubh.2020.571364

**Published:** 2020-11-24

**Authors:** Dara J. Lundon, Nihal Mohamed, Anna Lantz, Heather H. Goltz, Brian D. Kelly, Ashutosh K. Tewari

**Affiliations:** ^1^Department of Urology, Icahn School of Medicine at Mount Sinai Hospitals, New York, NY, United States; ^2^The Center for Scientific Diversity, The Icahn School of Medicine at Mount Sinai, New York, NY, United States; ^3^The Tisch Cancer Institute, The Icahn School of Medicine at Mount Sinai, New York, NY, United States; ^4^Department of Medical Epidemiology and Biostatistics, Karolinska Institutet, Stockholm, Sweden; ^5^College of Public Service, University of Houston-Downtown, Houston, TX, United States; ^6^Department of Urology, Austin Health, Melbourne, VIC, Australia

**Keywords:** COVID-19, SARS-CoV-2, social determinants of health, multi-ethnic, outcomes

## Abstract

**Importance:** The COVID-19 pandemic exploits existing inequalities in social determinants of health (SDOH) in disease burden and access to healthcare. Few studies have examined these emerging disparities using indicators of SDOH.

**Objective:** To evaluate predictors of COVID-19 test positivity, morbidity, and mortality and their implications for inequalities in SDOH and for future policies and health care improvements.

**Design, Setting, and Participants:** A cross sectional analysis was performed on all patients tested for COVID-19 on the basis of symptoms with either a history of travel to at risk regions or close contact with a confirmed case, across the Mount Sinai Health System (MSHS) up until April 26th 2020.

**Main Outcomes and Measures:** Primary outcome was death from COVID-19 and secondary outcomes were test positivity, and morbidity (e.g., hospitalization and intubation caused by COVID-19).

**Results:** Of 20,899 tested patients, 8,928 tested positive, 1,701 were hospitalized, 684 were intubated, and 1,179 died from COVID-19. Age, sex, race/ethnicity, New York City borough (derived from first 3 digits of zip-code), and English as preferred language were significant predictors of test positivity, hospitalization, intubation and COVID-19 mortality following multivariable logistic regression analyses.

**Conclusions and Relevance:** People residing in poorer boroughs were more likely to be burdened by and die from COVID-19. Our results highlight the importance of integrating comprehensive SDOH data into healthcare efforts with at-risk patient populations.

## Introduction

The novel coronavirus 2019 (COVID-19) caused by the SARS-CoV-2 virus, has triggered twin crises in public health and healthcare, resulting in over 18.8 million confirmed cases and 350,000 deaths globally. COVID-19 was first confirmed in the United States on January 31st 2020 in Washington State. Outbreaks of COVID-19 were subsequently reported in California and New York.

Published data characterize the resulting COVID-19 disease as one exploiting existing inequities in social determinants of health (SDOH) (1). The World Health Organization (WHO) defines SDOH as the conditions in and under which individuals are born, grow, work, and live, and the broader set of forces and systems (e.g., political, social, and economic policies and systems, social norms, and societal institutions) that shape the conditions and quality of daily life (2). U.S. Black/African Americans and Hispanic/Latinx are more likely to be diagnosed and experience COVID-19-related morbidities and mortality, especially those living in poor and crowded housing conditions, having pre-existing health comorbidities, low/limited incomes, or “essential” occupations (3). Data also reveal rapidly increasing inequities in disease burden among other minorities including Native American/American Indians (NA/AI) (3). Yet, few studies have examined these emerging inequities using indicators of SDOH (4). This study examines associations between COVID-19 test positivity, morbidity, and mortality and potential indicators of SDOH including patient- and neighborhood level variables and discusses implications of these drivers of health inequities for policies, research, and healthcare improvements.

## Materials and Methods

All patients who underwent testing for SARS-CoV-2 across the Mount Sinai Health System (MSHS) from March 28, 2020 to April 26th 2020 were included in this cross-sectional analysis (*N* = 20,913). Those with incomplete data were excluded (14 without a documented sex), or categorized as “Other/Unknown” (1,745 with unknown Race/Ethnicity, three for who zip-code/NYC borough was absent), or “Not asked” (669 were documented as “not-asked” for smoking status). SARS-CoV-2 testing was performed in MSHS through respiratory specimens that were evaluated by real-time reverse transcription polymerase chain reaction (RT-PCR) methods. Testing was performed on patients who had fever or signs/symptoms suggestive of respiratory illness and either a history of travel to affected areas (China, Japan, Italy, South Korea, and Iran), or close contact with a confirmed case of COVID-19 infection in the prior 2 weeks.

The MSHS Ethics Committee approved a waiver of documentation of informed consent; de-identified patient data was obtained from the MSHS Data Warehouse (https://msdw.mountsinai.org/). Data included demographics, behavioral, and clinical variables. In our analysis SDOH were defined in line with the 2017 WHO definition (2). Similar to Gottlieb et al. researchers selected specific sociodemographic characteristics within the database as proxies for SDOH, which have well-known significance within the social/behavioral science literature, to “translate” patient-level data into population-level data. Specifically, we used a consensus-building process involving our team of health disparities researchers, biostatisticians, and medical experts to develop these proxies of SDOH and health disparities in the existing dataset (e.g., age, gender, race/ethnicity, borough derived from first three digits of zip code of patient's place of residence, smoking status, and English as a preferred language; each of which are discussed further below). As suggested by Bazemore et al., we also utilized census information from the American Community Survey (ACS) to introduce neighborhood-level information such as median income by NYC borough (5, 6). The resulting SDOH proxies and markers of health disparity were included in models used in the current study.

We compared characteristics of patients according to the following outcomes: SARS-CoV-2 RT-PCR result (tested positive) for all patients who presented for testing, and of those who tested positive: hospitalization, intubation and mortality. Data were summarized as medians (interquartile range) for continuous variables and frequency (%) for categorical variables, where appropriate. We tested for bivariate associations between demographic, behavioral, and clinical variables using Chi-square (χ^2^) tests for categorical variables and Wilcoxon Rank Sum tests for the continuous variable “Age.” Patient characteristics are summarized in Table 1.

**Table 1:** Characteristics and bivariate analysis of patients with COVID-19 in this cohort.

		**Overall population**	**Test positivity**		**Hospitalization**		**Intubation**		**COVID-19 Mortality**	
		***n* = 20,899**	***n* = 8,928**	***P***	***n* = 1,701**	***P***	***n* = 684**	***P***	***n* = 1,179**	***P***
**Age, mean (*****SD*****)**		52.7 (20.6)	58.0 (18.8)	<0.001	58.2 (19.1)	<0.001	62.2 (18.2)	<0.001	72.9 (13.8)	<0.001
**Sex**, ***N*** **(%)**				<0.001		<0.001		<0.001		<0.001
	Male	10,488 (50.2%)	4,849 (46.2%)		997 (9.5%)		429 (4.1)		705 (6.7)	
	Female	10,411 (49.8%)	4,079 (39.2%)		704 (6.8%)		255 (2.4)		474 (4.6)	
**Race/ethnicity**, ***N*** **(%)**				<0.001		<0.001		0.461		0.008
	African ancestry	4,697 (22.5%)	2,191 (46.6%)		459 (9.8%)		164 (3.5%)		303 (6.5%)	
	White	6,294 (30.1%)	2,220 (35.3%)		392 (6.2%)		154 (2.4%)		334 (5.3%)	
	Asian	1,240 (5.9%)	429 (34.6%)		58 (4.7%)		33 (2.7%)		56 (4.5%)	
	Hispanic/Latinx	4,262 (20.4%)	2,210 (51.9%)		473 (11.1%)		186 (4.4%)		269 (6.3%)	
	Other/unknown	4,406	1,878 (42.6%)		319 (7.2%)		147 (3.3%)		217 (4.9%)	
**New York City borough**, ***N*** **(%)**				<0.001		<0.001		<0.001		<0.001
	The Bronx	1,513	602 (39.8%)		97 (6.4%)		23 (1.5%)		85 (5.6%)	
	Manhattan	9,259	3,731 (40.3%)		587 (6.3%)		247 (2.7%)		731 (7.9%)	
	Brooklyn	4,566	2,204 (48.3%)		473 (10.3%)		200 (4.4%)		155 (3.4%)	
	Staten Island	217	66 (30.4%)		5 (2.3%)		3 (1.6%)		8 (3.8%)	
	Queens	3,465	1,813 (52.3%)		455 (13.1%)		193 (5.6%)		137 (4%)	
	Long Island	477	140 (29.4%)		22 (4.7%)		5 (1%)		14 (2.9%)	
	Other	1,402	372 (26.5%)		62 (4.4%)		13 (0.9%)		49 (3.5%)	
**English as preferred language** ***N*** **(%)**				<0.001		0.4		<0.001		<0.001
	Yes	18,232	7,309 (40.1%)		1,379 (7.6%)		506 (2.8%)		885 (4.9%)	
	No	2,667	1,619 (60.7%)		322 (12.1%)		178 (6.7%)		294 (11%)	
**Smoking status** ***N*** **(%)**				<0.001		<0.001		0.948		0.280
	Yes	1,936	451 (23.3%)		91 (4.7%)		35 (1.8%)		46 (2.4%)	
	Passive	26	7 (24.9%)		3 (9.5%)		1 (5.0%)		1 (4.6%)	
	Previous smoker	4,812	2,067 (42.9%)		502 (10.4%)		172 (3.6%)		328 (6.8%)	
	Never	13,460	6,053 (45%)		1,040 (7.7%)		432 (3.2%)		684 (5.1%)	
	Not asked	665	351 (52.8%)		65 (9.8%)		43 (6.5%)		120 (18.1%)	
**Comorbidities** ***N*** **(%)**										
	Asthma	1,087	403 (4.5%)	<0.001	102 (6.0%)	<0.001	28 (4.1%)	0.652	45 (3.8%)	0.247
	Chronic obstructive pulmonary disease	597	232 (2.6%)	0.059	74 (4.4%)	<0.001	27 (3.9%)	0.029	67 (5.7%)	<0.001
	Hypertension	4,639	2,333 (26.1%)	<0.001	604 (35.5%)	<0.001	243 (35.5%)	<0.001	485 (41.1%)	<0.001
	Obesity	1,244	631 (7.1%)	<0.001	170 (10.0%)	<0.001	55 (8.0%)	0.343	90 (7.6%)	0.457
	Diabetes	3,159	1,675 (18.8%)	<0.001	436 (25.6%)	<0.001	182 (26.6%)	<0.001	325 (27.6%)	<0.001
	Chronic kidney disease	1,446	773 (8.7%)	<0.001	206 (12.1%)	<0.001	75 (11.0%)	0.030	188 (15.9%)	<0.001
	HIV	388	140 (1.6%)	0.009	42 (2.5%)	<0.001	6 (0.9%)	0.177	16 (1.4%)	0.619
	Cancer	2,333	674 (7.5%)	<0.001	166 (9.8%)	<0.001	41 (6.0%)	0.125	95 (8.1%)	0.521

*Categorical variables are presented as frequencies (%), and age is presented as mean with standard deviation. Borough of residence is presented as percentage of column for the overall population and percentage of row for each of the outcomes (test positivity, hospitalization, intubation, and COVID-19 mortality). We tested for bivariate associations between demographic, behavioral, and clinical variables using Chi-square (χ^2^) tests for categorical variables and Kruskal-Wallis tests for continuous variables*.

Multivariable logistic regression was performed for each of the above listed outcomes including all of the listed variables to control for potential confounding; as each co-variate was considered to be of clinical and/or social relevance. Statistical analyses were performed using R Statistical Software (type I error rate of 0.05) (7).

## Results

The study population consisted of 20,899 patients who underwent testing for SARS-CoV-2; of whom 8,928 tested positive, 1,701 were hospitalized, 684 were intubated, and 1,179 died from COVID-19. Median age in the overall population was 54 years and 50.2% were male. Patient's place of residence was distributed as follows: 44.3% Manhattan, 21.8% Brooklyn, 16.6% Queens, 7.2% The Bronx, 2.3% Long Island, 1.0% Staten Island, and 6.7% “Other.”

In univariate analysis nearly all evaluated predictors were significantly associated with the four Covid-19 outcomes. Positive test status was significantly associated with patients who were older, male, racial/ethnic minorities, current smokers, non-primary English speakers, and had comorbidities (e.g., asthma, hypertension, and diabetes; See Table 1). Patients living in Queens had significantly higher test positivity than those testing positive from all other boroughs in the MSHS catchment area. Hospitalization results mirrored those of test positivity, with notable exceptions. COVID-19-positive patients with chronic obstructive pulmonary disease (COPD) and those living in the Bronx and Manhattan were significantly more likely to be hospitalized; non-primary English speakers were not (Table 1). Once hospitalized, the COVID-19-positive patients who were significantly more likely to be intubated, were older; male; non-primary English speakers; residents of Queens; and had COPD, hypertension, diabetes, or chronic kidney disease. Those COVID-19-positive patients who died were significantly more likely to be older; male; White; former smokers; residents of Brooklyn and Queens; and comorbid with COPD, hypertension, diabetes, and chronic kidney disease (Table 1).

Multivariable regression analyses are presented in Table 2 and shows the adjusted odds of each of the outcomes: increasing age and male gender are significantly associated with higher odds of each outcome. Those of African ancestry are at significantly higher risk of each outcome compared to White race. In the adjusted analysis predicting test positivity, Hispanic/Latinx, and Other/Unknown race were associated with significantly higher odds of testing positive as compared to those of White race, but not mortality from COVID-19.

**Table 2:** Odds ratios presented after multivariable logistic regression analyses of patients who presented to MSHS; controlling for each of the listed variables: social determinants of health (SDoH), co-morbidities and other risk factors.

		**Test positivity**	**Hospitalization**	**Intubation**	**COVID-19 Mortality**
		**OR [95% CI]**	**OR [95% CI]**	**OR [95% CI]**	**OR [95% CI]**
**Age**		1.03 (1.02–1.03)	1.02 (1.02–1.02)	1.02 (1.02–1.03)	1.07 (1.07–1.08)
**Sex**					
	Male	Ref.	Ref.	Ref.	Ref.
	Female	0.70 (0.66–0.74)	0.81 (0.74–0.88)	0.69 (0.61–0.78)	0.69 (0.62–0.77)
**Race/ethnicity**					
	White	Ref.	Ref.	Ref.	Ref.
	African ancestry	1.70 (1.56–1.85)	1.47 (1.31–1.64)	1.39 (1.17–1.66)	1.28 (1.10–1.50)
	Asian	0.96 (0.84–1.10)	0.99 (0.81–1.20)	0.81 (0.59–1.11)	0.84 (0.63–1.12)
	Hispanic/Latinx	1.89 (1.73–2.07)	1.29 (1.14–1.46)	1.20 (0.99–1.45)	1.09 (0.92–1.30)
	Other/unknown	1.40 (1.28–1.52)	1.04 (0.92–1.18)	1.42 (1.19–1.69)	1.08 (0.91–1.27)
**New York City borough**					
	Manhattan	Ref.	Ref.	Ref.	Ref.
	The Bronx	1.06 (0.94–1.20)	0.79 (0.67–0.92)	1.12 (0.85–1.47)	1.07 (0.8–1.42)
	Brooklyn	1.49 (1.38–1.61)	0.38 (0.34–0.43)	1.62 (1.38–1.90)	2.06 (1.79–2.38)
	Staten Island	0.84 (0.68–1.04)	0.64 (0.47–0.86)	1.56 (1.01–2.39)	0.62 (0.32–1.18)
	Queens	1.02 (0.75–1.39)	0.76 (0.50–1.14)	1.03 (0.48–2.22)	1.87 (1.01–3.45)
	Long Island	1.63 (1.5–1.77)	0.46 (0.40–0.52)	1.64 (1.39–1.93)	2.21 (1.91–2.56)
	Other	0.73 (0.64–0.83)	0.82 (0.69–0.97)	2.09 (1.63–2.67)	1.18 (0.87–1.62)
**English as preferred language**					
	Yes	0.65 (0.59–0.72)	0.92 (0.81–1.04)	0.67 (0.57–0.79)	0.81 (0.70–0.94)
**Smoking status**					
	Never	Ref.	Ref.	Ref.	Ref.
	Passive	0.59 (0.21–1.69)	3.18 (1.21–8.39)	1.57 (0.20–12.19)	3.89 (0.97–15.54)
	Previous smoker	0.75 (0.69–0.82)	1.08 (0.97–1.20)	1.05 (0.89–1.25)	0.97 (0.84–1.13)
	Yes	0.33 (0.29–0.38)	0.96 (0.82–1.13)	1.21 (0.94–1.55)	0.91 (0.70–1.17)
	Not asked	0.98 (0.91–1.06)	0.84 (0.76–0.94)	1.60 (1.38–1.87)	1.31 (1.13–1.52)
**Comorbidities**					
	Asthma	0.82 (0.72–0.94)	0.90 (0.75–1.07)	0.76 (0.56–1.04)	0.68 (0.51–0.91)
	Chronic obstructive pulmonary disease	0.74 (0.62–0.90)	1.10 (0.90–1.36)	1.02 (0.74–1.4)	1.08 (0.84–1.40)
	Hypertension	0.96 (0.88–1.05)	1.07 (0.96–1.20)	1.38 (1.17–1.63)	1.18 (1.02–1.36)
	Obesity	1.42 (1.25–1.62)	1.17 (1.00–1.37)	1.24 (0.98–1.58)	1.39 (1.11–1.73)
	Diabetes	1.22 (1.11–1.34)	1.20 (1.07–1.34)	1.38 (1.17–1.64)	1.13 (0.97–1.31)
	Chronic kidney disease	1.02 (0.90–1.15)	1.34 (1.16–1.54)	1.11 (0.9–1.37)	1.55 (1.31–1.83)
	HIV	0.87 (0.69–1.09)	1.00 (0.77–1.3)	0.76 (0.46–1.25)	1.37 (0.91–2.05)
	Cancer	0.40 (0.36–0.45)	0.85 (0.75–0.96)	0.64 (0.51–0.79)	0.74 (0.62–0.88)

*The adjusted odds ratios (OR) are presented for testing positive for SARS-CoV-2 for all patients in this study, and adjusted odds ratios for being hospitalized, being intubated and mortality from COVID-19 are presented for those who tested positive for SARS-CoV-2*.

*SD, Standard deviation; OR, Odds ratio; CI, 95% confidence interval*.

Patients resident in Brooklyn, Long Island and Queens in NYC had a higher risk of death from COVID-19 on adjusted analysis as those resident in Manhattan (OR = 2.06 [1.79–2.38], 2.21 [1.91–2.56], and 1.87 [1.01–3.45], respectively, see Table 2). While residents of Brooklyn and Long Island had significantly higher risk of testing positive, and being intubated than those resident in Manhattan, residents from Queens did not have a higher risk of testing positive. Adjusting for each of the other social determinants and co-morbidities as listed in Table 2, those who speak English as their preferred first language have a significantly lower risk of testing positive, being intubated and death from COVID-19 than those who do not speak English as their preferred language. Comorbidities such as hypertension, obesity and chronic kidney disease were significantly associated with a higher risk of mortality from COVID-19 on adjusted analyses, while those diagnosed with COPD, diabetes and HIV were not. Those with a diagnosis of asthma had significantly lower risk of a positive test and mortality from COVID-19 while those with a cancer diagnosis had significantly lower risk of each outcome (Table 2).

**Figure 1:**
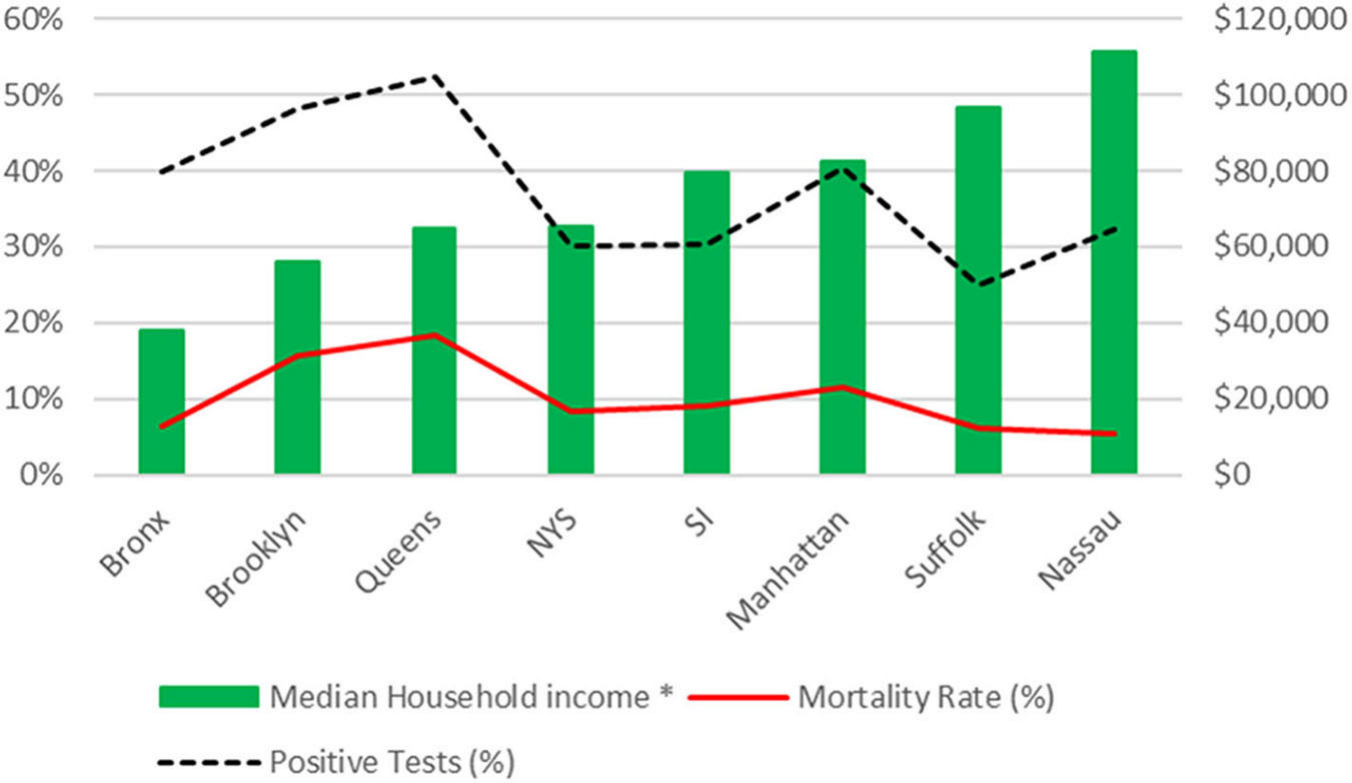
Test positivity rate (%) and mortality rate (%) from this cohort plotted vs. median household income by Zip Code. SI, Staten Island; NYS, New York State. *Median household income data as per 2018 U.S Census data.

There is an inverse relationship between mortality rate and the median household income for the borough from which patients reside (Figure 1), where lower median income corresponds with higher mortality rate.

## Discussion

COVID-19 continues to transform daily life for patients and those involved in patient care, highlighting health disparities and further confirming the link between SDOH and health outcomes. Our study is the first to demonstrate an impact of multiple indicators of SDOH and health disparities on COVID-19 test positivity, morbidity, and mortality outcomes in a patient population. We describe demographic, clinical, socioeconomic, and behavioral predictors of COVID-19 test positivity, morbidity, and mortality, at a time when more than 30% of all the US cases of COVID-19 were in New York (4). Our results demonstrate that each of the 6 SDOH and health disparities indicators examined in this study contributed to statistically significant worse outcomes for each of the study endpoints. This work highlights the importance of considering SDOH when caring for patients during the COVID-19 pandemic.

Our novel finding supporting the growing role of SDOH in the SARS-CoV-2 pandemic is that zip code (place of residence) and English as preferred first language is predictive of COVID-19 outcomes. Borough of residence has previously been described as showing strong associations with health literacy and income (8). Preferred first language is indicative of acculturation and is also a marker of health practices (9, 10). Prior research on infectious diseases pandemics has demonstrated that SDOH-related inequities create conditions for disease transmission and unequal burdens of disease morbidity and mortality (11). The significance of neighborhood environments and their relationship to heath maybe due to clustering of poverty with other forms of disadvantage. Figure 1 demonstrates that lower median income corresponds with higher mortality rate. While the relationship between a social determinant of health such as zip-code, and median household income is easier to examine and conceptualize, the relationship is more complex. There are multiple relevant constituents to consider for each individual social determinant we examined in this study; some which may overlap. Age, sex, race/ethnicity have been addressed in the context of COVID-19 outcomes, where older, male and African-American and Hispanic/Latinx patients have been documented to have worse outcomes (4, 12–16). Furthermore, there is a known association between smoking prevalence and location of residence in New York City (17). Smoking status itself is a health behavior which has been shown to magnify health disparities in groups with low incomes and without employment (18). An example of the potential overlap of this indicator of SDOH (i.e., zip code) and medical co-morbidities is the presence of respiratory conditions; for example asthma, which is exacerbated by pollution and allergens which are more common in disadvantaged neighborhoods. Other potentially relevant factors which could contribute to this finding relating to zip code/ place of residence, include food insecurity, commute times, educational attainment, housing density, number of persons per household, race/ethnicity, proximity to healthcare, occupation and healthcare literacy, which are all relevant components affecting how place of residence may impact health outcome (5, 19–22). There are complex relationships between social factors and health and so the causal roles of some social factors are not without controversy (23).

In line with prior studies, our findings provide insight into the intersectional, and unequal, effects of SDOH on COVID-19 testing, morbidity, and mortality, and highlight a need for including SDOH in COVID-19 studies as a means of contextualizing and interpreting such data (24). The COVID-19 pandemic is highlighting that one's zip code matters more than one's genetic code (25). In light of our findings, an index combining all potential patient- and community-level COVID-19 vulnerability indicators would be a valuable tool. Such a tool could guide healthcare and public health efforts to identify clinically- and community-modifiable targets for intervention. To facilitate development, public health and healthcare researchers and practitioners will need to work in tandem to integrate SDOH in electronic medical records (EMR) for use in such analyses, while also quickly implementing policies and procedures to enhance standardized collection of this data (26). While there is much literature describing the overlap between SDOH and co-morbidities, there are those who advocate that SDOH are themselves co-morbidities, and if they were considered as such, this could facilitate their broader documentation and collection (27).

This study has limitations including a study population drawn from one metropolitan area in the Northeast U.S. and de-identified data collected from an EMR database. First, generalizability may be low since trends in data from New York City and state may not mirror those in other affected areas (e.g., New Orleans, Chicago). Additionally, proximity to the MSHS facilities may have created a selection bias that influenced surveillance and outcomes data (e.g., positive tests were unequally represented across boroughs based on socioeconomic status). However, a strength of this study is that it is drawn from a region with uniform criteria for testing and likewise with uniform restrictions and regulations imposed to reduce the spread of COVID-19. Further, given that EMR are not designed with SDOH data collection as a primary objective, the latter represents a challenge. Study researchers could not include a precise, comprehensive set of SDOH in the analyses due to issues with balancing privacy and analysis in de-identified datasets for analysis (e.g., personal income). Further, complex SDOH indicators such as health literacy or neighborhood-level factors may have ICD-10 Z codes or require combinations of several *Z* codes. However, healthcare organizations may lack standardized measures for collecting this data; and codes may be imprecise in representing specific SDOH or inconsistently utilized in healthcare encounters. Thus, there is underrepresentation of SDOH in EMR (e.g., neighborhood-level social and environmental factors), and lack of uniform utilization of existing ICD-10 Z codes for complex SDOH. Future studies linking SDOH and clinical characteristics reflected in patient population's unique social and economic experiences (e.g., community norms and functioning, housing density, and food deserts) are needed to draw more stringent associations between SDOH and potential COVID-19 patient and subgroup outcomes among medically and socially vulnerable groups (e.g., smoking history and mortality).

## Conclusion

To our knowledge, this cross-sectional analysis represents the first large-scale analyses of multiple SDOH and indicators of health disparity in COVID-19 surveillance and clinical outcomes among patients in the New York City metropolitan area. Our results demonstrate differences in outcome based not just on race/ethnicity, age, gender, median household income, and borough of residence, but also preference of English as a first language.

This study highlights the importance of integrating comprehensive SDOH data into public health and healthcare efforts with at-risk patient populations and communities to improve the quality of COVID-19 prevention, surveillance, management, and policies.

## Data Availability Statement

The data analyzed in this study is subject to the following licenses/restrictions: Dataset remains the property of Mount Sinai Data Warehouse: msdw.mountsinai.org. Requests to access these datasets should be directed to msdw.mountsinai.org.

## Ethics Statement

The studies involving human participants were reviewed and approved by Mount Sinai Hospital IRB. Written informed consent from the participants' legal guardian/next of kin was not required to participate in this study in accordance with the national legislation and the institutional requirements. The MSHS Ethics Committee approved a waiver of documentation of informed consent as de-identified patient data was utilized in this study.

## Author Contributions

DL, HG, NM, and AT: study conception and design. DL, BK, and AT: acquisition of data. HG, AL, NM, and DL: drafting the manuscript. BK and AT: revising the manuscript critically for important intellectual content. All authors: analysis and interpretation of data. All persons who meet authorship criteria are listed as authors and all authors certify that they have participated sufficiently in the work to take public responsibility for the content, including participation in the concept, design, analysis, writing, or revision of the manuscript.

## Conflict of Interest

The authors declare that the research was conducted in the absence of any commercial or financial relationships that could be construed as a potential conflict of interest.
